# Optically addressable molecular spin qubits

**DOI:** 10.1557/s43577-026-01071-5

**Published:** 2026-04-09

**Authors:** Sarah K. Mann, Sam L. Bayliss

**Affiliations:** James Watt School of Engineering & Advanced Research Centre, University of Glasgow, Glasgow, G12 8QQ UK

**Keywords:** Optical, Quantum materials, Quantum sensing, Qubit, Sensor, Spin

## Abstract

Optically addressable molecular spins are a promising qubit platform, combining the chemical versatility, atomistic tunability, nanoscale modularity, and structural diversity of molecules with the coherence, detection sensitivity, and remote connectivity of optically readable spins. Here, we review progress developing and deploying optically readable molecular spin qubits and the key opportunities they present, with a particular focus on quantum sensing. We survey key criteria for realizing optically addressable spin qubits in molecules; discuss existing and emerging platforms—spanning coordination complexes, organic molecules, and both ground and excited-state spins; and outline emerging applications, open challenges, and opportunities for combining the richness of chemical systems with the power of optically readable spin qubits.

**Graphical abstract:**

**Figure:**
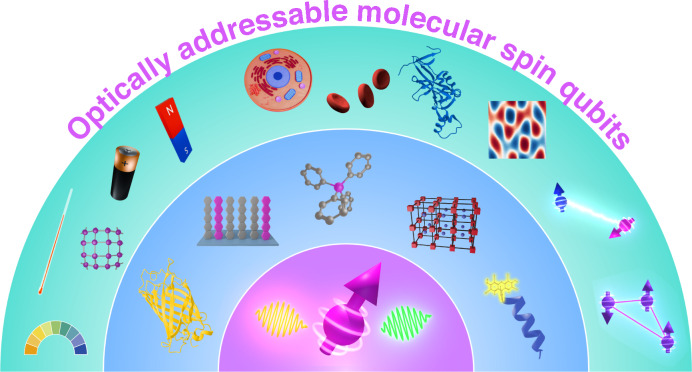
Optically addressable molecular spins as chemically versatile qubits.

## Introduction

Combining the coherence and control afforded by spins, with an optical interface for efficient initialization, sensitive and high-spatial-resolution readout, and long-range coupling, optically addressable spins have proven to be a powerful qubit platform for quantum science and technology. The opportunities of such a combination of properties are exemplified by solid-state color centers (i.e., semiconductor-based defects) such as the nitrogen-vacancy (NV) center in diamond and a growing class of other defect/host combinations.^1–10^ Optical-spin interfaces enable, for example, convenient and contactless room-temperature single-spin detection, and remotely interfacing qubits through photonic interconnects, facilitating numerous breakthroughs. For example, for quantum sensing, enabling nanoscale nuclear magnetic resonance^11,12^ and optical-magnetic imaging of biological systems^13^ and novel materials/devices,^14–16^ while for quantum networks, enabling kilometer-scale entanglement distribution.^17^

**Figure 1:**
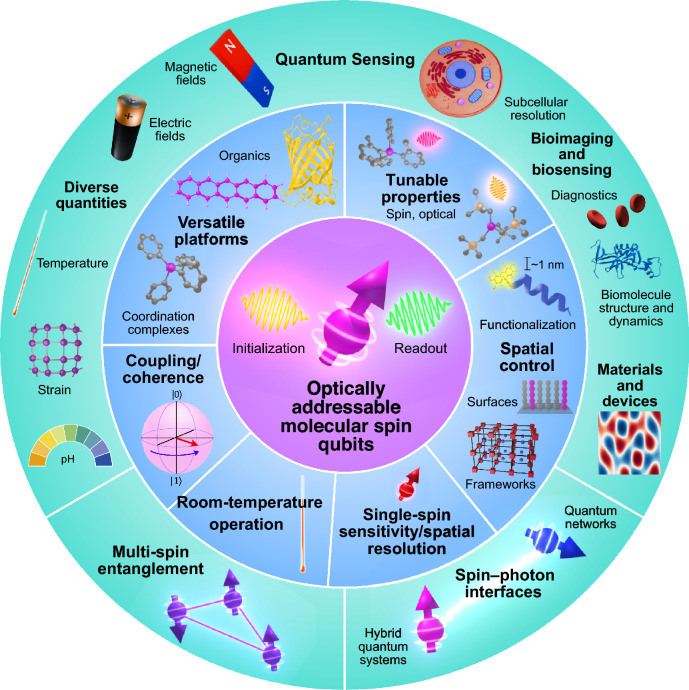
Overview of optically addressable molecular spin qubits, highlighting core functionalities (inner ring) and emerging or potential applications (outer ring).

**Figure 2:**
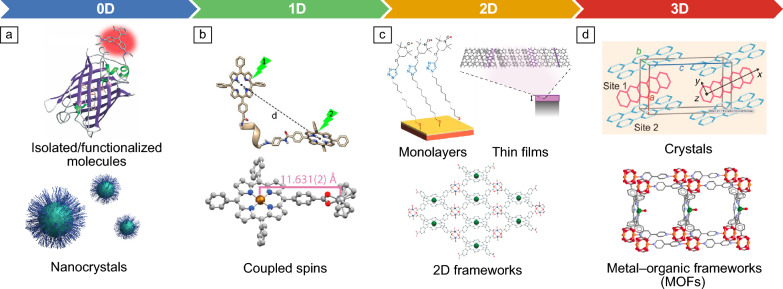
Overview of molecular spin qubit architectures. Credit: Panels adapted with permission from: (a) (Top) Reference 18, © 2017 Royal Society of Chemistry; (b) Reference 19 under a Creative Commons license CC BY 4.0, Reference 20, © 2021 American Chemical Society; (c) Reference 21 under a Creative Commons license CC BY 4.0; Reference 22 under a Creative Commons license CC BY 4.0; Reference 23, © 2018 Wiley; (d) Reference 24, © 2023 Wiley; Reference 25, © 2018 American Chemical Society.

Complementing the “top-down” nature of semiconductor-based platforms, molecular systems offer a “bottom-up” approach to quantum science/technology,^26–32^ with pioneering demonstrations from single-emitter detection^33,34^ and optically detected single-spin resonance of individual organic molecules,^35,36^ to electron spin coherence times approaching 1 ms^37^ and the creation of multi-spin architectures.^20,38–40^ These foundations, alongside the diverse domains molecular emitters/spins underpin—for example, fluorescence microscopy,^41^ single-photon sources,^42^ magnetic-resonance probes,^43^ and light-emitting diodes^44^—highlight compelling opportunities for optically addressable molecular spin qubits (OAMSQs; **Figure** 1) in which the enabling functionality of color centers meets the bottom-up control afforded by molecules. (See also References 31, 32, 45, 46 for complementary reviews focusing on molecular quantum sensing, coordination complexes for quantum networks, and optical interfaces to molecular and nonmolecular spins.)

Key opportunities of OAMSQs include: (1) atomistic tunability—enabling control over electronic/physical structure (e.g., emission wavelengths, placement of individual atoms) and spin properties (e.g., coupling strengths to external fields) for specific use cases (e.g., biosensing); and (2) spatial control—deploying molecules’ nanoscale size and modular nature, rich processing methods (e.g., solution-based, thermal evaporation, melt-reflow) and diverse architectures (e.g., individual molecules, nanocrystals, monolayers, thin films, porous frameworks,^47^ or DNA-based assemblies,^48–50^ **Figure** 2)—for proximal integration with other systems and the realization of multi-qubit structures.

As we describe in further detail below, such functionality offers unique opportunities for quantum sensing such as unprecedented nanoscale proximity to targets and enhanced couplings to nonmagnetic quantities such as temperature, strain, and electric fields; as well as novel opportunities to realize entangled multi-spin systems through bottom-up assembly, and tunable components for quantum networks and fundamental physics tests.

A central challenge for developing OAMSQs has been achieving the requisite optical spin readout capabilities but this has seen remarkable progress and here, we survey key recent developments and opportunities of OAMSQs, specifically focusing on systems that support optical initialization and readout. We outline key criteria and optical-spin interfaces for OAMSQs; survey current platforms—from coordination complexes to organic molecules—and key demonstrations; and outline opportunities, challenges, and emerging applications.

## Key criteria

OAMSQs should support:^51^ (1) a well-defined qubit, (2) optical spin initialization, (3) optical spin readout, and (4) coherent spin manipulation. As canonical two-level systems, molecular electron and nuclear spins are natural qubits that can be coherently manipulated through electron spin resonance (ESR) and nuclear magnetic resonance (NMR). Combined with optical readout, optically detected magnetic resonance (ODMR) becomes possible—enabling spin-resonance detection down to the single-molecule level^35,36,52^—and underpinning many applications of optically addressable spin qubits. For OAMSQs, combining a coherent spin with efficient optical initialization and readout then becomes key.

**Figure 3:**
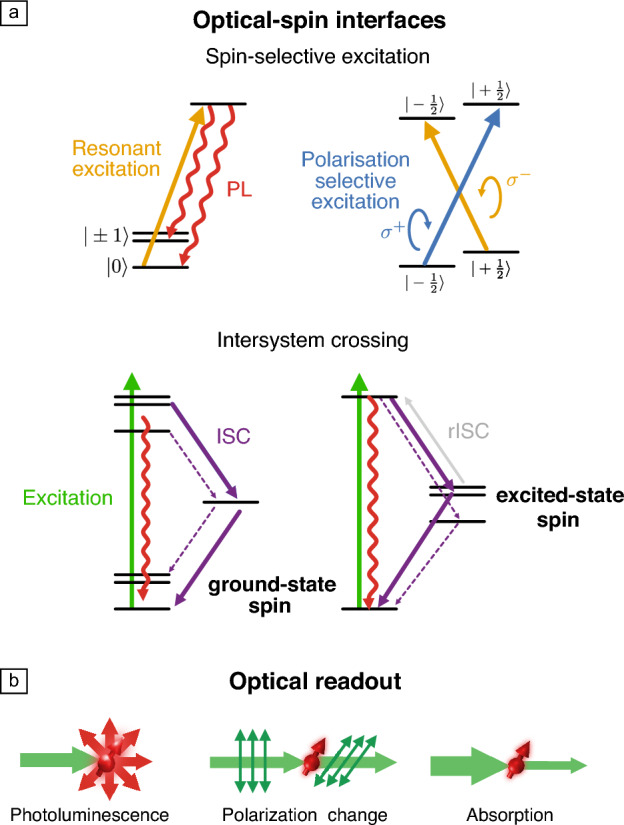
Optical-spin interfaces for qubit initialization and readout (a), and detection methods (b). PL, photoluminescence; ISC, intersystem crossing; rISC, reverse intersystem crossing.

### Optical spin initialization and readout

Optical spin initialization and readout mechanisms typically use either: (1) spin-selective excitation, or (2) spin-dependent intersystem crossing (**Figure** 3a), with detection typically through photoluminescence (PL)—discriminating between spin states by differences in emitted light intensity—but changes in polarization or absorption can also be used (Figure 3b).

#### Spin-selective excitation

In this approach, one spin-state is preferentially excited, shelving it in a different state and thereby generating spin polarization (either in the ground or excited state). Spin-selective excitation can be achieved through either a spin-dependent optical transition energy—enabling a specific spin sublevel to be excited based through its unique resonant excitation energy—or a spin-dependent optical transition polarization—enabling a specific spin sublevel to be excited based on its unique optical polarization selection rules (Figure 3a). Optical spin readout can proceed through PL, since only excited spin sublevels will emit; or alternatively, polarization or intensity changes of a probe beam (i.e., Faraday rotation^53^ or absorption). Resonant excitation offers high-fidelity initialization/readout, but requires narrow optical linewidths for spin selectivity, typically mandating cryogenic temperatures, while polarization-selective excitation is operable at room temperature, but requires suitable polarization selection rules.

#### Intersystem crossing

This approach to optical initialization and readout uses an optical cycle where transition rates between electronic states of different total spin (e.g., $S=0$ singlet, and $S=1$ triplet states) depend on which spin sublevel is involved (Figure 3a). Such intersystem crossing (ISC) dynamics are operable at room temperature and enable initialization through preferentially populating specific spin sublevels; and PL-based readout by creating differences in effective brightness between sublevels. This ISC mechanism can be mediated by spin-orbit or spin-spin (dipolar/exchange/hyperfine) interactions; is applicable for both ground and excited-state spins; and both forward and reverse ISC (rISC) processes can be used.^54,55^

## Platforms

The catalog of OAMSQs has expanded rapidly recently, spanning inorganic (i.e., metal-ligand coordination complexes) and organic (carbon- and hydrogen-based) molecules, including platforms based on ground and excited spin states. Here, we survey existing platforms and their unique features.

### Coordination complexes

**Figure 4:**
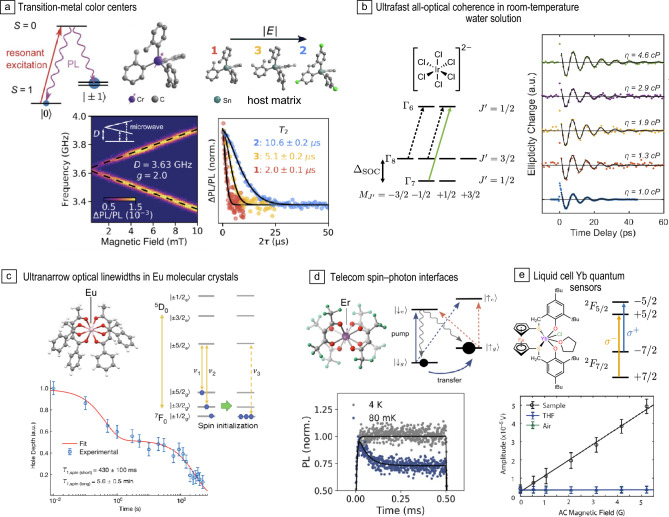
Coordination complexes with optically addressable ground-state spins. (a) Tunable Cr^4+^ molecular color centers: (Left) An optical-spin interface enables optically detected magnetic resonance (shown as a function of magnetic field and microwave frequency). (Right) Host-matrix tuning of zero-field splitting to enhance coherence. (b) Ultrafast all-optical coherence of [K_2_IrCl_6_] in room-temperature aqueous solution enables viscosity sensing by time-resolved Faraday ellipticity measurements. (c) Ultranarrow optical linewidths in a Eu^3+^ complex enable optical addressability of nuclear spins and nuclear-spin lifetime measurements. (d) Spin–photon interface at telecommunications wavelengths in an Er^3+^ complex enabling ground-state optical spin pumping at sub-Kelvin temperature. (e) Ultranarrow linewidths in a Yb^3+^ complex are leveraged to sense AC magnetic fields in a liquid environment. PL, photoluminescence, SOC, spin–orbit coupling. Credit: Panels adapted with permission from: (a) (Left) Reference 56, AAAS, (right) Reference 57 under a Creative Commons license CC BY 4.0; (b) Reference 58, AAAS; (c) Reference 59, Springer Nature; (d) Reference 60 under a Creative Commons license CC BY 4.0; (e) Reference 61, AAAS.

#### Transition metals

Transition-metal complexes support ground-state spins through the unpaired electrons of their partially filled *d*-orbitals, while offering tunability over spin and optical properties through their coordinating ligands and choice of metal center. Tremendous progress has been made with (non-optically addressable) transition-metal based molecular spin qubits—for example, $\simeq$1 ms electron-spin $T_2$-times at low temperatures^37^ and $\simeq$μs electron-spin $T_2$-times at room temperature^62^—however, optically interfacing such systems had remained elusive until recently.

***Chromium***—Optical addressability was achieved with $S=1$ ground-state spins in Cr^4+^ aryl complexes^56^ (diluted in a Sn-complex host matrix), which support optical initialization and readout through resonant excitation, alongside coherent microwave manipulation, serving as molecular color centers (**Figure** 4a, left). Varying the coordinating ligands enabled modification of zero-field splittings and optical transition energies,^56,63^ while modifying the Sn-complex host matrix enabled noise-insensitive “clock” transitions to be engineered to enhance spin coherence to $T_2\simeq 10\,\mu \mathrm{s}$^57^ (Figure 4a, right), highlighting the tunability and modularity offered by OAMSQs.

***Nickel, Vanadium, Molybdenum***—Ni^2+^,^64–66^ V^3+^,^67,68^ and Mo^3+^ ^68^ $S=1$ complexes have also been explored as analogous OAMSQs to the above chromium systems with Ni^2+^ offering air and water stability and therefore potential biocompatibility for quantum sensing; V^3+^ supporting optical transitions close to the telecommunications band for integration with fiber-optic quantum networks alongside an intrinsic nuclear spin ($I={7}/{2}$) as a potential quantum memory;^69^ and the increased spin–orbit coupling of Mo^3+^ offering promise for enhanced coupling to electric or strain fields. While these complexes have yet to demonstrate direct optical spin readout, they highlight the future potential for transition-metal based OAMSQs.

***Iridium***—As a distinct approach to optically interfacing transition metals, in 2024, Sutcliffe et al. used an air- and water-stable iridium complex—potassium hexachloroiridate(IV) (K_2_IrCl_6_)—to demonstrate how fast, spin-orbit- induced spin–lattice relaxation/dephasing can be countered by ultrafast all-optical measurements, enabling the detection of electron spin coherence in room-temperature solution (Figure 4b). The authors used polarization-selective optical excitation to create spin polarization in the complexes’ effective spin-1/2 ground-state from excited-state shelving, and monitored its evolution through the Faraday effect on a probe pulse. This enabled $T_2^\star$ times of $\simeq 10\,\textrm{ps}$ to be measured: $\sim$10^4^ times shorter than accessible with ESR. Furthermore, the authors achieved viscosity sensing from changes in $T_2^\star$.^58^ Subsequent work using $[\textrm{IrBr}_6]^{2-}$ achieved $T_2^\star \simeq 120\,\mathrm{ps}$ through matrix immobilization, and optical transitions in the biological window.^70^ While not affording full coherent manipulation, and short coherence times limit sensing interrogation times, these results highlight the opportunity of polarization-based room-temperature optical-spin interfaces.

#### Lanthanides

Lanthanides are attractive OAMSQs as—in addition to supporting ground-state spins—their 4*f* orbitals are shielded by filled outer 5*s*/5*p* orbitals, offering spectrally narrow 4*f*-4*f* optical transitions. Impressive demonstrations with (nonmolecular) lanthanide-based color centers—for example, single-ion detection in $\textrm{Pr}^{3+}$ ^71,72^ and $\textrm{Ce}^{3+}$;^73,74^ single-ion cavity coupling in $\textrm{Er}^{3+}$,^7^ $\textrm{Nd}^{3+}$,^75^ and $\textrm{Yb}^{3+}$; 6 h nuclear-spin coherence in $\textrm{Eu}^{3+}$ ^76^ and ensemble-based photonic quantum memories^77,78^—motivate lanthanide-based OAMSQs, with unique potential for chemical tunability, modular photonic integration, and construction of multi-spin systems.

***Europium***—Kumar et al. reported optical initialization and readout of ground-state nuclear spins in a binuclear $\textrm{Eu}^{3+}$ molecule^79^ through hole burning on the $^7{F}_0 - \, ^5D_0$ optical transition. Serrano et al. extended this work to a mononuclear $\textrm{Eu}^{3+}$ complex, demonstrating optical coherence times of $T_2\simeq 10\,\upmu \textrm{s}$ (corresponding to a linewidth equivalent of $\Gamma _h=1/(\pi T_2)\simeq 30\,\textrm{kHz}$); optical initialization of the $I=5/2$ ^151^Eu nuclear spin with $\simeq$95% spin polarization;^59^ and hole-recovery experiments revealing bi-modal nuclear-spin–lattice relaxation times of $\sim$0.4 and $300\,\textrm{s}$ (Figure 4c). The authors further used the atomic frequency comb protocol^80^ to store and retrieve an optical pulse in the spin ensemble. Additionally, they showed how interactions between “target” and “control” molecules could be realized by optically exciting the control molecule, whose photo-induced electric field shifts the optical transition of the target molecule. Subsequent studies explored hole burning of other $\textrm{Eu}^{3+}$ complexes, paving the way for further synthetic enhancements,^81,82^ and recently optically detected nuclear magnetic resonance was achieved.^83^ Related work has demonstrated photonic-cavity enhanced $\textrm{Eu}^{3+}$-complex emission^84,85^ highlighting opportunities to externally engineer optical-spin interfaces.

***Erbium***—Fiber-optic losses are minimized around 1530–1565 nm (the telecom C-band), making emitters at these wavelengths attractive for long-range quantum networking. In addition, such wavelengths are compatible with mature silicon photonics. $\textrm{Er}^{3+}$, with $\simeq$1550 nm optical transitions has therefore attracted interest, with impressive demonstrations such as single-shot spin readout achieved with (nonmolecular) crystal defects.^86^ Recently, a spin-optical interface to molecular $\textrm{Er}^{3+}$ has been demonstrated (Figure 4d)^87^ showcasing a coherently controllable effective spin-1/2 ground state and—through resonant excitation of the $\simeq$1530 nm $J=15/2$-$J=13/2$ transition—spin-resolved optical transitions with $\simeq$11 MHz homogeneous linewidths, and optical spin pumping at sub-Kelvin temperatures.

***Ytterbium***—Molecular Yb^3+^ complexes support coherent spin manipulation^88–91^ and narrow optical transitions.^92^ Shin et al. recently demonstrated ytterbium complexes with particularly narrow ensemble optical linewidths of $\simeq$150 GHz in room-temperature solution alongside homogeneous optical linewidths of $\simeq$100 kHz at 77 K.^61^ The narrow room-temperature linewidths enabled magnetic-circular-dichroism-based measurements of magnetic fields with an AC sensitivity of $\simeq 3\,\upmu \textrm{T}/\sqrt{\textrm{Hz}}$ (Figure 4e) and the authors outlined opportunities for such systems to serve as solution-based analogs of atomic vapor cells with $\sim$10^6^ higher number densities, which could enable reduced sensor-sample distances.

### Organic molecules

Organic molecules’ weak spin–orbit coupling and capacity for high-oscillator strength optical transitions makes them attractive for combining long spin lifetimes—even at room temperature—with bright emission, and their rich tunability and established applications—from LEDs and photovoltaics to biomicroscopy—are attractive for synergistically developing OAMSQs.

**Figure 5:**
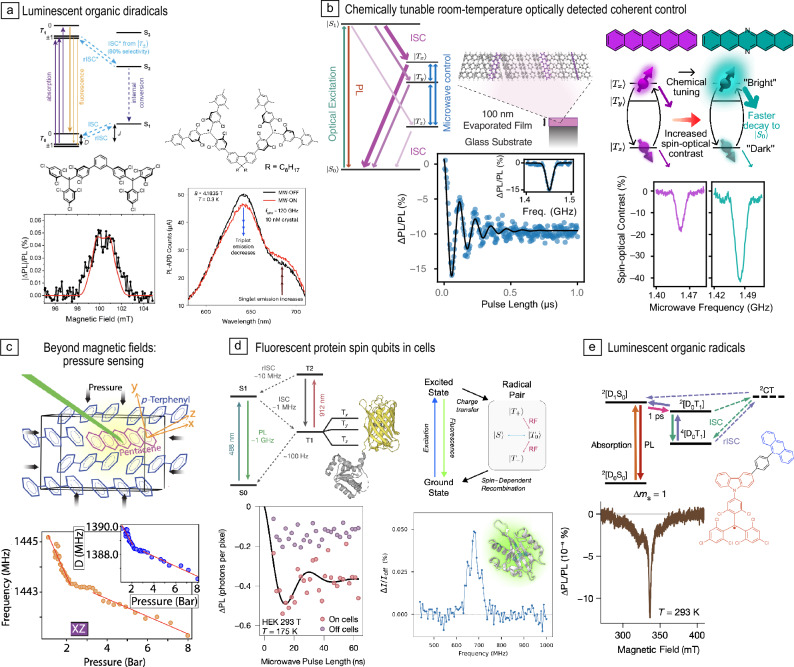
Optically addressable organic spins. (a) Luminescent organic diradicals as optically addressable ground-state molecular qubits. (b) Chemically tunable room-temperature optically detected coherent control of molecular spins in organic chromophores: (Left) Energy-level diagram for photoexcited triplets illustrating the spin-selective intersystem crossing (ISC) optical-spin interface; optically detected Rabi oscillations of a pentacene doped *para*-terphenyl thin film. (Right) Pulsed optically detected magnetic resonance (ODMR) spectrum of pentacene and diazapentacene demonstrating enhanced optical-spin contrast due to modified spin dynamics. (c) Pressure sensing in a pentacene doped *para*-terphenyl crystal. (d) Fluorescent protein spin qubits functioning in cells: (Left) Enhanced yellow fluorescent protein and optically activate delayed fluorescent readout scheme, enabling in-cell optically detected coherent control. (Right) Fluorescent protein spin qubit (AsLOV2) functioning in living cells. (e) A luminescent organic-radical chromophore platform with a spin-optical interface, enabling room-temperature ODMR of a photogenerated quartet spin. PL, photoluminescence, rISC, reverse intersystem crossing. Credit: Panels adapted with permission from: (a) (Left) Reference 93, American Chemical Society; (right) Reference 94 under a Creative Commons license CC BY 4.0; b (left), Reference 22 under a Creative Commons license CY BY 4.0; (right), Reference 95 under a Creative Commons license CY BY 4.0; (c) Reference 96 under a Creative Commons license CC BY 4.0; (d) (left) Reference 54, Springer Nature; (right) Reference 97 under a Creative Commons license CY BY-NC 4.0; (e) Reference 55 under a Creative Commons license CY BY 4.0.

#### Ground-state spins

Most emissive organic molecules comprise closed-shell spin-0 ground states. However, important exceptions offer potential for optically addressable ground-state spins.

***Emissive radicals/diradicals***—While organic radicals (i.e., open-shell $S \ge 1/2$ molecules) are often unstable or weakly emissive, there are important exceptions. In particular, polychlorinated triarylmethyl radicals are stable and highly emissive; motivating growing interest in LED applications and wider spin/optical functionality.^44,98–102^ By linking two radicals to form a diradical, a triplet ground state can be formed, and therefore a similar level structure to the NV center. Poh et al. outlined the theoretical design principles for luminescent organic diradicals supporting a triplet ground state and ISC-based initialization/readout,^103^ and to enhance ODMR contrast.^104^ Kopp et al. demonstrated ODMR at 80 K from a phenyl-linked TTM-based diradical^93^ (**Figure** 5a, left) and Chowdhury et al. demonstrated optical spin initialization/readout^94^ in a fluorene-bridged diradical, achieving $\sim$8% ODMR contrast at 300 mK (Figure 5a, right), with emission from both triplet and singlet manifolds, and near-unity photoluminescence quantum yields. Parallel studies demonstrated magneto-luminescence in emissive diradicals,^105,106^ and Kopp et al. demonstrated optically detected coherent spin control at 85 K in a toluene-bridged TTM diradical, with chemically tuned ODMR contrast.^107^ Recent work from Chowdhury et al.^108^ showing near-unity photoluminescence yields, ODMR and a large ($\sim$10%) magneto-PL in room-temperature diradicals indicates promise for ambient operation. Diradicals’ brightness, spin lifetimes, and tunability hold promise for future applications while challenges include weak ground-state inter-radical exchange and dipolar interactions—populating both triplet and singlet ground states at room temperature, and making zero-field manipulation more challenging.

***Carbenes***—Carbenes comprise a divalent carbon atom (forming only two covalent bonds with neighboring groups) leaving two (predominantly) nonbonding electrons that can form a triplet ground state (e.g., Hund’s rule predicts the two degenerate *p*-orbitals in a linear carbene would favor a triplet ground state).^109,110^ Pioneering early work demonstrated hole burning on carbene triplet-triplet fluorescence excitation spectra at 2 K, with spin-dependent optical transitions from differences in ground/excited-state zero-field splittings, alongside evidence for ground-state spin polarization through ISC.^111^ Recent work theoretically analyzed how carbenes could realize high-fidelity optical-spin interfaces,^112^ and Roggors et al. experimentally demonstrated ODMR in 2,2'-dinaphthylcarbene (DNC), achieving >40% ODMR contrast, coherent spin control, and $T_2 = 157\,\upmu$s at 5 K in a perdeuterated crystal.^113^ While promising ongoing efforts continually improve carbene stability,^114–116^ their high reactivity typically makes them only stable at cryogenic temperatures and are classically generated *in situ* by photolysis from their diazo precursor^117^ making higher-temperature applications challenging. Photogeneration presents an opportunity, however, for photopatterning spins which has found utility for color centers.^118^

***Nitrenes***—The nitrogen-analogue of carbenes, nitrenes can support ground-state spin triplets^119^ and potentially optical-spin addressability. While nitrene optical-spin interfaces have yet to be shown, recent work demonstrating nitrenes that are stable, and emissive in crystals at room-temperature;^120,121^ alongside computational guidelines for nitrene optical-spin addressabillity^122^ highlight future potential.

#### Excited-state spins

Photogenerated spins can further serve as OAMSQs: in particular, photoexcited triplets formed from ISC from an excited singlet; and higher spin states generated through coupling these triplets to additional spins (triplets or radicals). While metastable spins potentially present tradeoffs (e.g., in lifetime) compared to ground-state spins, they are widespread, can be “turned off,” and have demonstrated key milestones.

***Photoexcited triplets: chromophores***—Optical-spin addressability of photoexcited triplets has a long history: from initial demonstrations of cryogenic ODMR enabled by ISC;^123–125^ through room-temperature ~μs coherence using conventional ESR;^126,127^ to pioneering single-spin ODMR of individual pentacene molecules at liquid-helium temperatures.^35,36^ Only recently however was room-temperature optically detected coherent spin manipulation demonstrated—using pentacene doped in *para*-terphenyl—providing a proof of principle of such functionality in molecules.^22,128^ The simplicity and ubiquity of molecules supporting photoexcited triplets, their bright fluorescence and room-temperature operability are attractive for future applications. Demonstration of room-temperature pulsed ODMR in thermally evaporated thin films (Figure 5b, left) highlight the deployment strategies available for OAMSQs,^22^ while chemical tuning has enabled room-temperature optical-spin contrast to reach 40% (Figure 5b, right)^95^—exceeding the typical 30% in NV centers. Recent work has also highlighted photoexcited triplets’ potential as probes of nonmagnetic quantities: (Figure 5c) with ~10^3^-times higher pressure coupling and larger temperature couplings in pentacene compared to NV centers.^96^ High-contrast room-temperature pulsed ODMR of molecular photoexcited triplets in nanocrystals^95,129^ demonstrates how the benefits of a crystalline environment (e.g., long $T_1$-times) can be retained, while affording molecular opportunities (e.g., high spin densities, chemically enhancing couplings, and flexible surface chemistry and solution-based fabrication). Ishiwata et al.’s recent demonstration of organelle-selective intracellular temperature sensing using pentacene-doped *para*-terphenyl nanocrystals functionalized with a biocompatible surfactant further highlights the biosensing potential.^129^ Further exciting opportunities for photoexcited triplets include all-optical spin manipulation for single-molecule photonic switches^130–132^ and interfacing nuclear-spin ensembles for matter-wave interferometry.^133^

***Fluorescent proteins***—Enabling deterministic tagging of biological targets through genetic encoding, fluorescent proteins have realized remarkable applications in optical microscopy of biological systems.^134^ Recently, multiple studies demonstrated metastable triplets in fluorescent proteins as OAMSQs.^54,135–137^ Using enhanced yellow fluorescent protein Feder et al.^54^ demonstrated ODMR using an innovative optically activated delayed fluorescence spin-readout mechanism (Figure 5d, left). At 80 K, they achieved 20% ODMR contrast and a $16\,\,\upmu \text {s}$ $T_2$ time under CPMG dynamical decoupling; further demonstrating coherent control in human embryonic kidney cells at 175 K alongside ODMR in *Escherichia coli* cells at room temperature. Several independent studies demonstrated ODMR of flavin-containing proteins,^135–137^ attributed to a radical-pair mechanism involving the protein backbone as the electron donor and the bound flavin cofactor as the acceptor. Abrahams et al.^135^ employed a variant of the AsLOV2 protein (MagLOV)—engineered via directed evolution to enhance its magneto-optical response^138^—achieving ODMR from a single living bacterial (*E. coli*) cell (Figure 5d, right), while Burd et al. demonstrated wide-field ODMR imaging in a living nematode (round worm) genetically modified to express a red fluorescent protein–flavin.^137^ Collectively, these studies open exciting possibilities for *in vivo* biosensing—because fluorescent proteins can be expressed directly in host organisms—and enhancing optical-spin interfaces through genetic engineering (e.g., via rational design^139^ or directed-evolution methods).^140^

***Radical pairs***—Beyond the previously discussed fluorescent proteins, photogenerated radical pairs present a broad class of both biological and synthetic systems that have been explored as quantum sensors in avian navigation,^141^ as synthetic qubits,^142^ and for organic spintronics and optically spatially mapping magnetic fields.^143–146^ Established ODMR and magneto-luminescence in such systems highlight potential for further developments as OAMSQs.

#### Higher spin states (S > 1)

Higher-spin systems provide potential opportunities for exploiting a larger Hilbert space or achieving enhanced coupling strengths, and several systems have demonstrated optical addressability.

***Chromophore-radical systems***—$S=3/2$ (quartet) states—generated by coupling a chromophore-generated triplet to a radical—have been explored through ESR as qubit platforms with room-temperature electron spin coherence^147^—see Reference 148 for a recent review. $S=2$ (quintet states) have also been realized by appending a chromophore with two radicals.^149–152^ Optical spin readout of such systems was only recently demonstrated however, using emissive radicals (TTM-1Cz) covalently linked to a chromophore (anthracene) (Figure 5e).^55^ Energetic resonance between doublet and triplet photoexcitations enabled quartet formation and, crucially, their fluorescence-based readout through reverse ISC, enabling room-temperature quartet ODMR (Figure 5e), with recent work further developing design principles.^153^ Gorgon et al. further showed how quartet-coupled emission could be realized with non-emissive radicals via coupling to thermally activated delayed fluorescence (TADF) chromophores whose small singlet-triplet splitting enables reverse ISC from the quartet to chromophore $S_1$ states, thereby opening a distinct class of luminescent quartets.^154^

***Singlet-fission***—Optically addressable $S=2$ quintet states can be formed through singlet-exciton fission (primarily explored for its potential to enhance solar-cell efficiency), where a photoexcited singlet state is converted into a pair of triplets on neighboring chromophores.^155,156^ These triplet pairs can evolve into a quintet and be coherently manipulated^157,158^—even at room temperature^159–161^ and optically read out through spin-dependent radiative recombination, enabling ODMR.^162–167^ Furthermore, quintet-forming dimers can be created,^158,160,168–170^ offering a modular two-chromophore unit for future applications.

## Opportunities

Having surveyed existing OAMSQ platforms, we now highlight key functional opportunities and emerging applications across quantum sensing, quantum networks, and multi-spin system creation.

**Figure 6:**
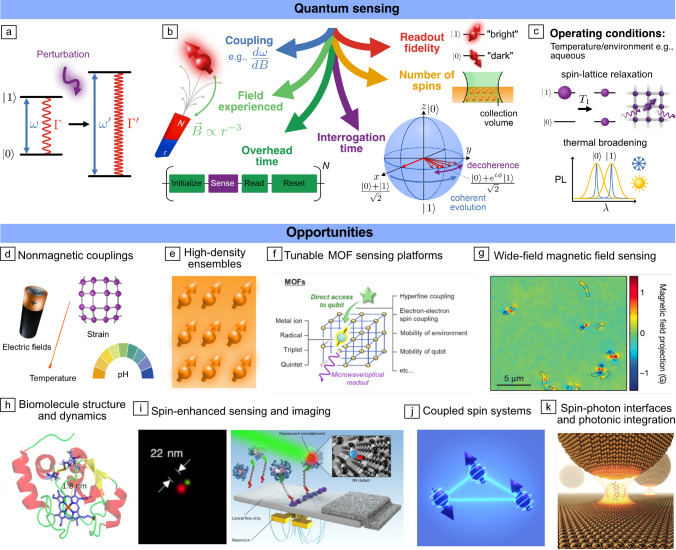
Opportunities and emerging applications of optically addressable molecular spin qubits. (a) Operating principle of spin-based quantum sensing: A perturbation modifies the spin transition frequency ($\upomega$) or relaxation rate ($\Gamma$), enabling its detection. (b, c) Key metrics for quantum sensing encompassing the sensitivity to an external field; the field that can be experienced; and the operating conditions. (d–k) Opportunities for optically addressable molecular spin qubits: (d) Couplings between spins and nonmagnetic quantities. (e) High-density ensembles. (f) Leveraging self-assembled porous frameworks. (g) Wide-field magnetic imaging (illustrated for nitrogen-vacancy [NV]-sensing of magnetotactic bacteria). (h) Spin-label based probing of biomolecules (illustrated for pulsed electron spin resonance distance measurements between a photoexcited porphyrin triplet and a nitroxide spin label on the protein cytochrome C). (i) Spin-enhanced sensing and imaging: (Left) Deterministic emitter switch microscopy for super-resolution imaging (illustrated with NV centers). (Right) Enhanced *in vitro* diagnostics (demonstrated in fluorescent nanodiamonds) where a microwave field modulates emission intensity to separate the signal from background autofluorescence. (j) Multi-spin entanglement. (k) Spin–photon interfaces and photonic integration. MOFs, metal–organic frameworks. Credit: Panels adapted with permission from: (f) Reference 47, American Chemical Society; (g) Reference 13, Springer Nature; (h) Reference 171, American Chemical Society; (i) (left) Reference 172, American Chemical Society; (right) Reference 173, Springer Nature; (k) Reference 174, Springer Nature.

### Quantum sensing

Spin-based quantum sensing uses changes in qubit energy levels or transition rates to detect a physical quantity (**Figure** 6a).^175^ For example, DC and AC energy-level shifts can be detected by measuring the phase accumulated during a Ramsey^176^ or dynamical decoupling sequence respectively, while transition-rate changes can be detected through changes in spin–lattice relaxation times (i.e., $T_{1}$-relaxometry, which can be implemented all optically). Key parameters governing quantum-sensor performance are the *combination* of: (1) sensitivity to the relevant physical quantity; (2) the actual external field that can be experienced by the spins (i.e., target proximity); and (3) their compatibility with relevant operating conditions (e.g., room temperature). While the sensitivity $\upeta$ dictates the minimum detectable field in a given time *T* ($X_{\text {min}}=\upeta / \sqrt{T}$), the actual sensing signal-to-noise ratio is determined by the ratio of the field experienced $X_{\text {actual}}$ to the minimum detectable field (i.e., $\text {SNR}=X_{\text {actual}}\sqrt{T}/\upeta$). Because $X_{\text {actual}}$ depends on sensor-target proximity (e.g., scaling with distance as $r^{-3}$ for magnetic-dipole coupling), it is therefore the *combination* of sensitivity and proximity that becomes key (Figure 6b), with the operating conditions dictating use cases.

#### Sensitivity

Sensitivity (i.e., the smallest resolvable signal in a given time) is determined by the factors outlined in Figure 6b, and—considering an energy shift and ODMR-based readout scheme for concreteness—scales (approximately) as:^177^

1 $$\begin{aligned} \upeta \propto \frac{1}{\upgamma C\sqrt{N n_{\text {avg}}}}\frac{\sqrt{t_{\text {o}}+T_{2}^{\chi }}}{T_{2}^{\chi }}, \end{aligned}$$

where $\upgamma$ is the coupling strength, that is, the energy shift per change in external quantity (e.g., for magnetic coupling in the linear Zeeman regime, $\upgamma =\Delta m_sg_e\upmu _B$, where $\Delta m_s$ is the change in spin projection, $g_e$ the electronic *g*-factor, and $\upmu _B$ the Bohr magneton); *C* is the optical-spin contrast; *N* the number of spins; $n_{\text {avg}}$ the average number of photons collected per spin per readout (i.e., the “brightness”); $t_{\text {o}}$ the measurement overhead time (required for initialization and readout); and $T_{2}^{\chi }$ is $T_2^\star$ for DC sensing and $T_2$ (or the equivalent under dynamical decoupling) for AC sensing and sets the interrogation time. These six parameters therefore situate opportunities and challenges for OAMSQ sensitivities which we now discuss before turning to proximity and operating conditions.

***Enhanced couplings***—A stronger coupling to the sensing field induces greater spin-phase accumulation in a given time and therefore an increased sensing signal. While molecular systems can enhance magnetic couplings (e.g., using transitions of high-spin systems or the large *g*-factors found in lanthanides^87^) a compelling opportunity is for enhanced coupling to *nonmagnetic* quantities such as temperature, electric fields, strain/pressure, pH or other quantities (Figure 6d). The utility of such spin-based sensing modalities has been elegantly demonstrated in color centers^178–180^ but with limited coupling strengths and scope for tuning. Through chemical control, OAMSQs could significantly exceed existing nonmagnetic couplings and therefore improve detection sensitivity. For example, optimized OAMSQs could advance nanoscale thermometry which has been powerfully demonstrated with NV centers^178^—offering insight into, for example, local metabolic processes in cells—but would benefit from higher sensitivities. Likewise, optimizing spin-strain couplings could realize novel sensors or faciltate quantum transduction between different degrees of freedom, while optimizing spin-electric coupling could enable nanoscale electrometry,^181^ localized electrical gating, and spin manipulation. Molecular demonstrations of spin-temperature couplings (at room temperature) of $\simeq$100 kHz/K^96^ (exceeding the $74\,\mathrm {kHz/K}$ of NV centers^182^); room-temperature spin-pressure couplings of $1.8\,\mathrm {MHz/bar}$ (~1000× the $1.5\,\mathrm {kHz/bar}$ of NV centers^183^); and spin-electric couplings of $11\,\mathrm {Hz/(V/m)}$^184^ (~65× the $0.17\,\mathrm {Hz/(V/m)}$ of NV centers^179^) highlight the potential for OAMSQs with unprecedented sensitivities to nonmagnetic quantities.

***Readout fidelity***—The combination of “brightness” ($n_{\text {avg}}$) and contrast (*C*) determines how effectively imparted changes can be detected. With $\upeta \propto C^{-1}n_{\mathrm{avg}}^{-1/2}$ (Equation 1), the contrast is particularly important. Notably room-temperature contrasts in excess of the typical 30% of NV centers have now been achieved for photoexcited triplets^95^—with promise for further tuning—while ~10 ns radiative lifetimes and near-unity PL quantum yields have been shown for diradicals^94^ (both similar to the NV center^185^). Combining high contrast and brightness will allow existing ensemble demonstrations to extend to single spins, thereby opening OAMSQ quantum sensors with nanoscale spatial resolution.

***Number of spins***—For ensemble applications, maximizing the number of spins (*N*) in a given volume increases the signal (as $\sqrt{N}$) without sacrificing spatial resolution. Their chemical creation makes OAMSQs attractive for achieving high spin densities (Figure 6e; for example, circumventing parasitic lattice damage, which leads to unwanted electronic noise sources in defects^186^). For example, molecular spin ensembles can support ~μs $T_2$-times at electron-spin densities of $\uprho \sim 10^{25}$/m^3^ (~10^4^ ppm concentration),^57^ in excess of the $\uprho \sim 10^{24}$/m^3^ used in high-density defect ensembles (~10 ppm NV concentration) with comparable coherence times.^187^ Beyond electron spins, the high-density and optically polarizable nuclear-spin bath of OAMSQs could find interesting applications (e.g., for analyzing objective collapse models of quantum mechanics).^133^

***Interrogation (coherence) time***—The coherence time, $T_{2}^{\chi }$ sets how long phase can be accumulated for and is typically dominated by the magnetic fields from surrounding nuclei (Figure 6b)—although other mechanisms such as librationally induced zero-field splitting modulations,^188^ spin-orbit mediated spin relaxation, and electronic spin–spin interactions at sufficiently high concentrations can play a role. Strategies to extend coherence in OAMSQs include reducing noise sensitivity through “clock” transitions;^57,189^ reducing nuclear-spin noise, for example, through deuteration,^37,190,191^ or developing nuclear-spin-free qubits;^192^ and implementing dynamical-decoupling protocols.^187^

***Overhead time***—Minimizing the ratio of the overhead time $t_\text {o}$ (required for initialization/readout) to the interrogation time (i.e., $\sqrt{t_\text {o}+T_{2}^{\chi }}/T_{2}^{\chi }$) is important for optimizing the acquired signal per unit time with opportunities through both chemically tuned ISC rates^95,193^ as well as optimized readout schemes.^54^

#### External field experienced (proximity)

The modular nature, ~1 nm size, and chemical assembly of OAMSQs promises a level of spatial control and proximity that is particularly attractive in maximizing the target field in quantum sensing. For example, reducing sensor-target distances from 10 to 2 nm yields an ~100-times increase in a magnetic-dipole field. While continuous advancements improve the coherence of near-surface NV centers, their coherence is typically significantly degraded when ~10 nm from the surface due to, for example, noise-inducing dangling bonds, highlighting opportunities for OAMSQs for which the “surface” chemistry is well defined. Such benefits parallel the motivation for spin qubits in 2D materials (e.g., hBN^194,195^), where even with modest coherence times from a dense nuclear-spin bath, proximal integration can afford benefits.^196^ The architectural diversity available with molecular systems offers multiple routes to such proximal integration (Figure 2): from functionalized individual molecules, surface layers (e.g., self-assembled monolayers and frameworks, solution-processed or evaporated thin films), and porous metal–organic frameworks^47^ (Figure 6f) opening up utility in, for example, biosensing, wide-field spin-based imaging (Figure 6g), and solid-state sensing of solution- or gas-phase analytes, respectively.

#### Biosensing

We now turn to a specific opportunity area of biosensing, drawing together opportunities outlined so far. OAMSQs with suitable chemical groups open up targeted integration into biological systems, as routinely deployed in fluorescence microscopy, and ESR-based spin-labeling. Bio-integrated OAMSQs hold promise to merge the established disciplines of molecule-based optical microscopy and spin resonance, realizing new opportunities for quantum biosensing. Recent demonstrations of in-cell optical spin readout of fluorescent proteins^54,135^ and molecular nanocrystals^129^ highlight the promise for such a direction and recent work integrating emissive radicals into biological systems through both nanoparticles^197–199^ and water-soluble derivatives,^200^ alongside the established use of related radicals as electron paramagnetic resonance (EPR) spin labels^201^ highlights scope for further systems to contribute. The ability to optimize OAMSQs’ couplings to nonmagnetic quantities such as temperature; tune emission into the biological transmission window (650–1350 nm^202^); enable multiplexing through distinct spin-optical responses;^47,54,135^ and draw on established techniques (e.g., for functionalization) from the purely optical or magnetic resonance communities highlights promising applications.

One specific opportunity of having a functionalizable, nanoscale, and co-localizable molecular spin system is to realize optically readable spin labels where the established EPR-based technique of using the interaction between two spins as a probe of structure and dynamics in biological systems could be combined with the sensitivity of optical detection to enable spin-label experiments on individual biomolecules (or much smaller ensembles than available with EPR-based detection). Recent developments using photoexcited triplets as EPR-detected spin labels highlights the potential of this direction (Figure 6h).^19,171,203^

As demonstrated with NV centers, the ability to deterministically switch emitters “on and off” through ODMR can enable super-resolution (i.e., sub-diffraction limit) optical microscopy because closely spaced emitters can be separately turned on and off if they have distinct spin resonance spectra (Figure 6i, left).^172^ Bringing such a spin-based super-resolution approach to molecular systems could provide a complementary deterministic approach to emitter switching compared to current all-optical super-resolution methods. Similarly, elegant demonstrations with the NV center have shown how deterministically modulating emission through ODMR can be used to reduce background signals in fluorescent assays, and therefore push their sensitivity limits for early-stage diagnostics (Figure 6i, right),^173^ applications which could be enhanced by molecules’ tunability and high-density operation.

Although isolated functionalized emitters are promising for minimizing proximity to targets, albeit with exposure to the environment, molecular nanocrystals could sacrifice proximity for environmental protection,^95,129,204^ while offering complementarity to diamond nanocrystals,^205^ through more controllable surface chemistry, higher emitter densities, and enhanced couplings to nonmagnetic quantities (e.g., temperature).

### Coupled spin systems

Synergistic with quantum sensing, OAMSQs provide unique opportunities for constructing interacting multi-spin systems by drawing on their capacity for bottom-up assembly. While remarkable demonstrations have been made using stochastically occurring nuclear or electron spins around defects,^206–208^ deterministic creation of coupled multi-qubit systems remains an open challenge. Such capabilities could enable deterministic proximal coupling of >2 separately addressable OAMSQs for multipartite entanglement, the creation of small processing units, and entanglement-enhanced sensing (Figure 6j), or the creation of interacting spin ensembles for probing many-body physics, which, through chemically assembled one-, two-, or three-dimensional architectures, could complement impressive demonstrations with defect ensembles.^209,210^

### Spin–photon interfaces and photonic integration

The tunability, modularity, and novel processing methods of molecular systems make them appealing for spin–photon interfaces, integration with photonic devices and the creation of quantum networks (Figure 6k). Impressive demonstrations coupling spin-0 transitions of organic molecules to photonic structures/cavities^42^ highlight opportunities to enhance spin–photon interfaces through molecular-enabled techniques. For example, melt-reflow crystal growth for photonic cavity integration,^42^ or self-assembly enabled plasmonic coupling.^174^ The tunability of OAMSQs further holds potential for spin–photon interfaces at telecom wavelengths (e.g., O-band 1260–1360 nm; C-band 1530–1565 nm), which are critical for long-range entanglement by minimizing photon loss in optical fibers; exploiting established infrastructure/components; or enabling integration with silicon photonics; as has been a key focus for defects. Demonstrations of vanadium ($\simeq$1240 nm)^67^ and erbium ($\simeq$1550 nm)^87^ OAMSQs highlight future potential, benefiting from the above photonic-coupling opportunities.

### First-principles design

The vast space of realizable OAMSQs is one of their key opportunities but also poses challenges for identifying and screening optimal targets and understanding environmental interactions. First-principles calculations offer an invaluable toolbox to understand, optimize, and predict improved properties for OAMSQs spanning spin coherence,^57,211^ spin–lattice relaxation,^212,213^ ISC dynamics,^104^ electronic and spin structure,^214,215^ optical transition rates and coherence,^216^ and host effects.^217^ With rapid feedback between theoretical prediction and experimental realization, the ability to leverage techniques and materials being developed in adjacent functional molecular materials domains, alongside growing molecular libraries; designing and optimizing tailor-made OAMSQs from first-principles holds exciting promise.

## Challenges

Here, we highlight some specific challenge areas for future work: operating temperature and environment, brightness, and optical coherence.

### Operating temperature

While exciting applications probing novel materials and devices are available at low temperatures,^218^ room-temperature operation is particularly desirable for its convenience and relevance to physiological environments, and has been a key enabler for defect-based applications. So far room-temperature OAMSQs include photoexcited triplets in organic chromophores^22,95,128^ and fluorescent proteins,^54,135^ alongside quartets in radical-coupled chromophores^55^ and quintets in singlet-fission systems.^166^ A key challenge for room-temperature operation of OAMSQs is achieving sufficiently long spin–lattice relaxation times, $T_1$ (Figure 6c) in relevant environments, which, through spin mixing, impacts optical spin readout and ultimately limits coherence. For OAMSQs exploiting spin-selective excitation on the other hand, the temperature dependence of optical linewidths is also key in defining the operating conditions (Figure 6c). For transition metal coordination complexes, achieving room-temperature operation (e.g., through a nonresonant optical-spin interface, and management of spin-orbit-induced spin–lattice relaxation) remains an outstanding challenge.

### Operating environment

While optical and spin properties are optimized under low-noise environments—for example, rigid solid-state hosts to prolong $T_1$, or nuclear-spin-free surroundings to prolong $T_2$—the qubit’s compatibility with the environment of interest determines its deployability, for example, remaining sufficiently photostable, and resilient to environmental noise to retain coherence and optical spin readout in aqueous solution for biological sensing. While remaining a key area for future work, encouraging progress includes room-temperature ODMR of photoexcited triplets in aqueous solution and living cells,^54,129,135^ and lanthanide complexes operating in room-temperature solutions.^58,61^

### Brightness

In some systems, achieving high optical brightness remains challenging. For transition metal coordination complexes, this arises from the low oscillator strengths of *d*–*d* transitions, while in lanthanide-based complexes, weak 4*f*–4*f* transitions lead to long optical lifetimes and correspondingly low emission rates. Integration with photonic structures—as demonstrated with Yb^3+^^219^ and Eu^3+^ complexes^84,85^—holds promise for future enhancements. Organic chromophores, although often bright, can be limited by their photostability.

### Optical coherence

Optical coherence (or linewidth) determines the efficacy of remote-entanglement protocols (through two-photon interference);^220,221^ the performance of photonic quantum memories;^80^ and the fidelity of spin-initialization/readout under resonant excitation. Ideally, the optical coherence would be set by the optical lifetime. While the lifetime limit has been achieved for spin-0 transitions in polyaromatic hydrocarbons,^42^ OAMSQs have not yet reached it. Eu complexes have displayed the longest absolute optical coherence of $\simeq 10\,\upmu \textrm{s}$ but with the tradeoff of long $\simeq 500\,\upmu \textrm{s}$ optical lifetimes.^59^ Reaching lifetime-limited lines (e.g., through engineering vibrational modes, reducing $T_{opt}$ through an optical cavity) remains an important direction for future work, as does all-optical coherent spin manipulation.^222,223^

## Conclusions

Overall, OAMSQs present an exciting emerging frontier for quantum science and technology in which bottom-up techniques can be combined with the demonstrable advantages of opticallly interfaced spins. While at an early stage, as outlined here, OAMSQs have seen great progress in the last years, including: optical-spin interfaces to transition-metal color centers, luminescent organic radicals/diradicals and carbenes; ultrafast coherence detection in room-temperature solutions; narrow optical linewidths and telecom spin–photon interfaces in lanthanide complexes; 40% room-temperature ODMR contrast in photoexcited triplets; and genetically encodable fluorescent protein spin qubits operating in living cells. While we have highlighted particular potential for quantum-sensing—as well as realizing multi-spin systems and spin–photon interfaces—as the field develops, we anticipate new applications to emerge that leverage the unique properties of a molecular approach, opening up exciting interdisciplinary advances through bespoke qubits optimized through a feedback loop across theory, synthesis, and measurement.
